# Luminol-conjugated cyclodextrin biological nanoparticles for the treatment of severe burn-induced intestinal barrier disruption

**DOI:** 10.1093/burnst/tkad054

**Published:** 2024-03-13

**Authors:** Yajun Song, Yang Li, Wengang Hu, Feng Li, Hao Sheng, Chibing Huang, Xin Gou, Jingming Hou, Ji Zheng, Ya Xiao

**Affiliations:** Department of Urology, Xinqiao Hospital, The Army Medical University, No. 184, Xinqiao Street, Shapingba District, Chongqing, 400037, China; Department of Urology, Xinqiao Hospital, The Army Medical University, No. 184, Xinqiao Street, Shapingba District, Chongqing, 400037, China; Department of Urology, The First Affiliated Hospital of Chongqing Medical University, No. 1, Yuanjiagang Road, Yuzhong District, Chongqing, 400016, China; Department of Urology, Xinqiao Hospital, The Army Medical University, No. 184, Xinqiao Street, Shapingba District, Chongqing, 400037, China; Institute of Burn Research, Southwest Hospital, State Key Laboratory of Trauma, Burn and Combined Injury, Chongqing Key Laboratory for Disease Proteomics, Army Military Medical University, No. 30, Gaotanyan Street, Shapingba District, Chongqing, 400038, China; Department of Urology, Chongqing University Three Gorges Hospital, No. 165, Xincheng Road, Wanzhou District, Chongqing, 404031, China; Department of Urology, Xinqiao Hospital, The Army Medical University, No. 184, Xinqiao Street, Shapingba District, Chongqing, 400037, China; Department of Urology, Xinqiao Hospital, The Army Medical University, No. 184, Xinqiao Street, Shapingba District, Chongqing, 400037, China; Department of Urology, The First Affiliated Hospital of Chongqing Medical University, No. 1, Yuanjiagang Road, Yuzhong District, Chongqing, 400016, China; Department of Rehabilitation, Southwest Hospital, The Army Medical University, No. 30, Gaotanyan Street, Shapingba District, Chongqing, 400038, China; Department of Urology, Xinqiao Hospital, The Army Medical University, No. 184, Xinqiao Street, Shapingba District, Chongqing, 400037, China; Department of Urology, Xinqiao Hospital, The Army Medical University, No. 184, Xinqiao Street, Shapingba District, Chongqing, 400037, China

**Keywords:** LCD nanoparticle, Burn, Intestinal barrier, Inflammatory cytokines, reactive oxygen species, Vγ4+ γδ T cells, Luminol-conjugated cyclodextrin

## Abstract

**Background:**

The breakdown of intestinal barrier integrity occurs after severe burn injury and is responsible for the subsequent reactions of inflammation and oxidative stress. A new protective strategy for the intestinal barrier is urgently needed due to the limitations of the traditional methods. Recently, the application of nanoparticles has become one of the promising therapies for many inflammation-related diseases or oxidative damage. Herein, we developed a new anti-inflammatory and antioxidant nanoparticle named luminol-conjugated cyclodextrin (LCD) and aimed to evaluate its protective effects in severe burn-induced intestinal injury.

**Methods:**

First, LCD nanoparticles, engineered with covalent conjugation between luminol and β-cyclodextrin (β-CD), were synthesized and examined. Then a mouse burn model was successfully established before the mouse body weight, intestinal histopathological manifestation, permeability, tight junction (TJ) expression and pro-inflammatory cytokines were determined in different groups. The proliferation, apoptosis, migration and reactive oxygen species (ROS) of intestinal epithelial cells (IECs) were assessed. Intraepithelial lymphocytes (IELs) were isolated and cultured for analysis by flow cytometry.

**Results:**

LCD nanoparticle treatment significantly relieved the symptoms of burn-induced intestinal injury in the mouse model, including body weight loss and intestinal permeability abnormalities. Moreover, LCD nanoparticles remarkably recovered the mechanical barrier of the intestine after severe burn, renewed TJ structures, promoted IEC proliferation and migration, and inhibited IEC apoptosis. Mechanistically, LCD nanoparticles dramatically alleviated pro-inflammation factors (tumor necrosis factor-α, IL-17A) and ROS accumulation, which could be highly involved in intestinal barrier disruption. Furthermore, an increase in IL-17A and the proportion of IL-17A^+^Vγ4^+^ γδ T subtype cells was also observed *in vitro* in LPS-treated Vγ4^+^ γδ T cells, but the use of LCD nanoparticles suppressed this increase.

**Conclusions:**

Taken together, these findings demonstrate that LCD nanoparticles have the protective ability to ameliorate intestinal barrier disruption and provide a therapeutic intervention for burn-induced intestinal injury.

## Graphical Abstract

**Graphical Abstract:**
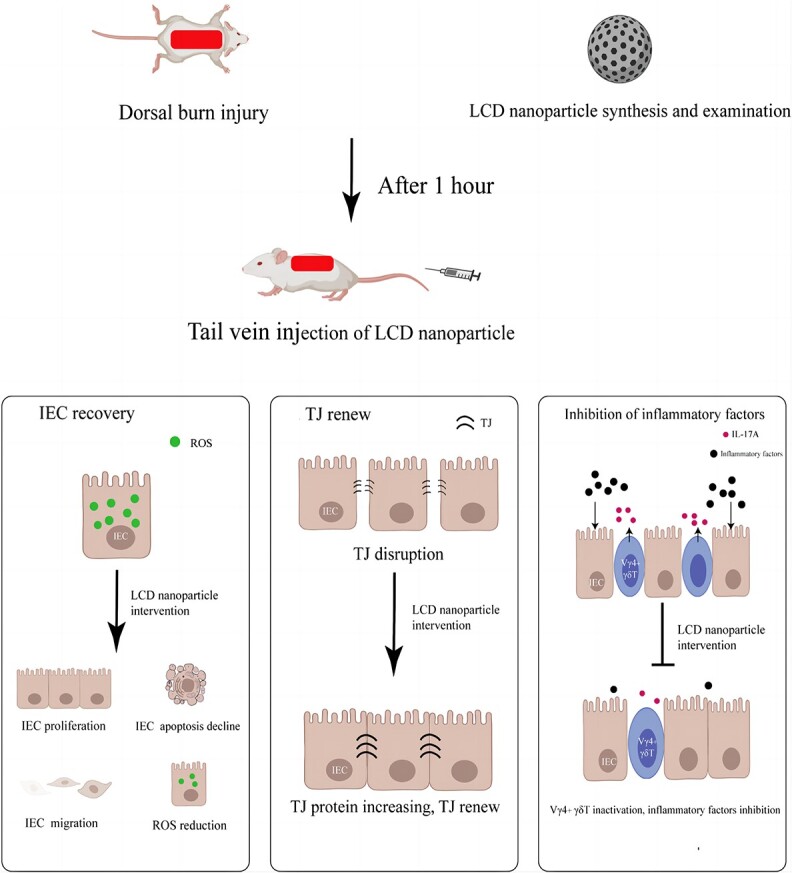

Highlights

- The breakdown of intestinal barrier integrity occurs after severe burn injury and is responsible for the reactions of inflammation and oxidative stress.
- A new anti-inflammatory and antioxidant nanoparticle named LCD was developed, and its protective effects of severe burn-induced intestinal injury were evaluated.
- LCD nanoparticles relieved the symptoms of burn-induced intestinal injury in a mouse model, and maintained the intestinal mechanical barrier through alleviating pro-inflammation factors (tumor necrosis factor-α, IL-17A) and ROS accumulation.
- LCD nanoparticles suppressed the increase in IL-17A and the proportion of IL-17A^+^Vγ4^+^ γδ T subtype cells that are considered important triggers in the early stage of inflammation.

## Background

Severe burn injury (>30% of the total body surface area) is a significant clinical problem worldwide [1,2]. This type of burn injury can not only cause deep skin tissue necrosis but can also increase the risks of sepsis, shock, multiple organ dysfunction syndrome or even death [3,4]. Previous studies have suggested that disruption of the intestinal epithelial barrier is closely related to these postburn diseases, and inflammatory reactions are one of the main culprits for the breakdown of the intestinal barrier [5]. Following the process of severe burn injury, intestinal inflammation and subsequent oxidative stress ensue, destroying the intestinal barrier via damaging intestinal epithelial cells and the tight junction (TJ) structures in between [6]. Massive death of epithelial cells and the downregulation of TJ proteins lead to increased intestinal permeability and microbial dysbiosis, which creates a favorable environment for opportunistic pathogens to pass through the bloodstream and cause systemic infection [7,8]. Later, systemic infection can trigger severe postburn diseases. Therefore, attenuating intestinal inflammation and reactive oxygen species (ROS) at an early stage may be a promising strategy for the treatment of postburn intestinal barrier disruption.

Interleukin (IL)-17A, a proinflammatory cytokine, plays a critical role in initiating and amplifying inflammatory responses [9–11]. IL-17A can induce the release of other proinflammatory cytokines, such as tumor necrosis factor-α (TNF-α), IL-1β and IFN-γ, and promote inflammation by recruiting neutrophils and macrophages [12,13]. Studies have shown that γδ T cells are the major source of IL-17A in the intestinal immune system. This γδ T-cell-derived IL-17A promotes an inflammatory response and damages the intestinal epithelium at the early stage of inflammatory bowel disease [14]. Furthermore, our previous studies indicated that the Vγ4^+^ γδ T subtype cells are the important lymphocytes that produce IL-17A in a burn injury intestinal model [15]. These studies concluded that Vγ4^+^ γδ T cells are likely to be the dominant producers of IL-17A in the early inflammatory response in the burn model.

**Figure 1:**
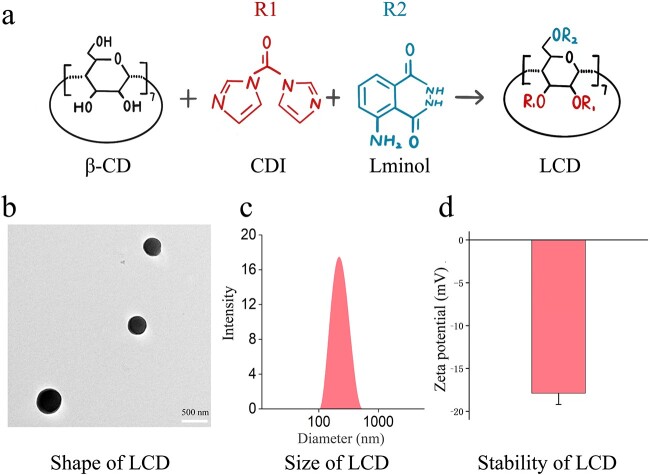
LCD engineered as anti-inflammatory nanoparticles. (**a**) Schematic illustration of the chemical structure of LCD. (**b**) TEM images of LCD nanoparticles, (**c**) size and (**d**) zeta potential of LCD nanoparticles. Scale bar: 500 nm. *β-CD* β-Cyclodextrin, *CDI* 1-carbonyldiimidazole, *LCD* luminol-conjugated cyclodextrin

There are no specific drugs for postburn intestinal barrier disruption. Nonsteroidal anti-inflammatory drugs and corticosteroids are broadly used for the different inflammatory diseases with severe complications, such as gastrointestinal diseases [16], nervous system risk [17] and allergy [18]. Recently, nanoparticle materials have been used as innovative and effective therapeutics against inflammatory diseases [19–21]. These desirable materials conjugated with anti-inflammatory agents can directly target sites and modulate inflammatory and oxidative reactions, with the advantages of reducing side effects and improving comfortable administration [22,23]. We have successfully developed a new and safe nanoparticle material, namely luminol-conjugated cyclodextrin (LCD) biological nanoparticles, which is formed by a cyclic oligosaccharide β-cyclodextrin (βCD) conjugated with luminol (Figure 1a). The interaction of luminol and β-CD effectively initiates anti-inflammatory mechanisms [24]. It has been proved that LCD nanoparticles effectively attenuate inflammation-induced diseases, including peritonitis, acute lung injury and atherosclerosis, and significantly inhibit the release of inflammatory cytokines and ROS by neutrophils and macrophages [25]. The decrease in the release of inflammatory factors may be attributed to T cells regulation [26]. Furthermore, we have also confirmed that LCD nanoparticles can hydrolyze into water-soluble products and then be excreted through the kidney, causing no adverse effects [25,27].

In the present study, we investigated whether the administration of LCD nanoparticles results in the protection of intestinal barrier integrity as well as a reduction in inflammatory factors (Figure 1b) and ROS. Moreover, we carried out a preliminary exploration of the possible mechanism of the anti-inflammatory effects of LCD nanoparticles . This study indicated a novel potential of LCD nanoparticles to treat burn-induced intestinal injury.

## Methods

### Materials

Luminol, β-CD, 1-carbonyldiimidazole (CDI), anhydrous dimethylformamide (DMF), ethylenediaminetetraacetic acid (EDTA), 1,4-dithio-D-threitol, diaminobenzidine, mercaptoethanol and FITC-dextran (4000 MW) were purchased from Sigma–Aldrich (St. Louis, USA). Anti-TNF-α, anti-IL-17A, anti-TGF-β, anti-zonula occludens-1 (ZO-1), anti-occludin and anti-ki67 were obtained from Invitrogen (Carlsbad, USA) and Biolegend (San Diego, USA). Flow cytometry monoclonal antibodies (mAbs) anti-CD3 mAb, anti-TCR γδ mAb and anti-TCR Vγ4 mAb were obtained from Abcam (Cambridge, UK) and Stemcell (Vancouver, Canada). The terminal deoxynucleotidyl transferase dUTP nick-end labeling (TUNEL) apoptosis assay kit, reactive oxygen species assay kit, cell counting kit-8 (CCK-8) and EasySep™ human gamma/delta T-cell isolation kit were purchased from Beyotime Biotechnology (Jiangsu, China) and Stemcell (Vancouver, Canada). Dulbecco’s modified Eagle’s medium (DMEM) and fetal bovine serum (FBS) were obtained from Invitrogen (Carlsbad, USA).

### Preparation of LCD nanoparticles

LCD nanoparticles were synthesized by chemical modification of β-CD. β-CD was first activated by mixing with CDI in anhydrous DMF solution. Then, activated β-CD was obtained by freeze-drying, and CDI–β-CD mixed with luminol was dissolved in anhydrous DMF. Finally, the luminol-conjugated β-CD compound was precipitated from deionized water. Then, LCD nanoparticles were identified and conformed. The characterization and shape were tested by transmission electron microscope (TEM) (HT7700, Hitachi, Japan), the average hydrodynamic diameter was calculated through dynamic light scattering (DLS) and the stability of particle dispersion was tested by electrophoresis.

### Animals

C57BL/6 mice (female, 6–8 weeks old, weighing 20–25 g) were supplied by the Animal Center of the Army Medical University. Mice were housed under standard conditions with free access to water and food, controlled temperature (25 ± 2°C) and 12-h light and dark cycles. They were acclimatized for 1 week before the studies. All experiments were approved by the Animal Care and Use Committee of the Third Military Medical University.

### Preparation of burn injury model and LCD nanoparticles treatment

The concentration of LCD nanoparticles was measured according to our previous experimental studies [25]. Mice were randomly divided into three groups, namely, the sham group, burn group, and burn + LCD group (200 mg/kg). Each mouse was injected with 4 mg of LCD nanoparticles. After an overnight fast, all mice were intraperitoneally anesthetized with sodium pentobarbital (40 mg/kg) and shaved from their dorsal area. The burn model was established according to the method previously used in our laboratory. Mice for the burn treatment were immersed in a 90°C water bath for 10 s, causing full-thickness third-degree burns involving 30% of the total body surface are, while control mice were immersed in 37°C water for 10 s. After these procedures, Ringer’s solution (50 ml/kg) was intraperitoneally injected for resuscitation. All mice were allowed to recover in equipment thermostated to 42°C to wake up from the anesthesia. After 1 h, mice in the burn + LCD group were given an intravenous injection of LCD nanoparticles, while mice in the other groups were treated with an equivalent dose of saline at the same time.

### Overall health assessment after severe burn injury

The weights of the mice were recorded every day. After 7 days, the mice were sacrificed, and the whole small intestine sections were dissected. Intestinal appearance, length and thickness were assessed.

### Assessment of severity of injury of distal ileum

A 0.5 cm section of the distal ileum was harvested for histopathological evaluation. For staining analysis, the tissue was first fixed in 4% buffered formalin. Then, samples were dehydrated in increasing grades of ethanol and embedded in paraffin for sectioning. Finally, intestinal sections were stained with hematoxylin and eosin (HE). The severity of intestinal injury was assessed by pathological scoring criteria in a blinded fashion. For transmission electron microscopy analysis, the tissue was fixed in precooled 3% glutaraldehyde solution overnight. The specimens were postfixed in 1% osmium tetroxide dissolved in saline and embedded in polyester resin, and then ultrathin sections were made. Intestinal sections were stained with lead citrate and uranyl acetate and observed under an electron microscope (HT7700, Hitachi, Japan).

### Intestinal permeability measurement

At 12 h after the procedure, the mice were anesthetized and laparotomized. A 5 cm segment of distal ileum was dissociated and tied at both ends by wool thread. A total of 0.2 ml of FITC-dextran (10 mg/ml) was injected into the ileum. Then, this segment was returned to the abdominal cavity and moist gauze was used to cover it. After 30 min, systemic blood was collected from the inferior vena cava and plasma was obtained from the blood by centrifugation. Plasma fluorescence was measured in a fluorescence spectrophotometer (Varioskan Flash, Thermo Fisher, USA) at a wavelength of 480 nm/520 nm.

### Immunohistochemistry and immunofluorescence staining

Intestinal sections were harvested and fixed in 4% buffered formalin. After deparaffinization and dehydration, the tissue sections were incubated in sodium citrate solution in a boiling water bath for antigen retrieval. Then, nonspecific binding sites were blocked with 5% goat serum. After that, the tissues were incubated with rabbit anti-TNF-α, anti-IL-17A, anti-TGF-β, anti-ZO-1, anti-occludin or anti-ki67 primary Abs at 4°C overnight. Horseradish peroxidase-labeled goat anti-rabbit secondary Ab was added. These tissues were stained with a diaminobenzidine substrate and contrasted using hematoxylin. Images were observed under a light microscope (BX53, Olympus, Japan). For immunofluorescence staining, using the TUNEL apoptosis assay kit, the apoptotic cells were observed and counted under a fluorescence microscope (BX53, Olympus, Japan).

### Immunoblotting analysis

Inflammatory cytokines and TJ protein expression were detected by western blotting. The distal ileum mucosa was collected and protein was extracted by RIPA lysis buffer with a cocktail of protease inhibitors. After the supernatant was obtained by centrifugation, protein was quantified using a standard BCA protein assay. Equal amounts of protein (25 μg) were fractionated on 8 and 12% sodium dodecyl sulfate–polyacrylamide gel electrophoresis gels and then transferred to polyvinylidene difluoride membranes. After the nonspecific sites were blocked with 5% skimmed milk, the membranes were incubated with Abs specific for TNF-α, IL-17A, TGF-β, ZO-1, occludin or β-actin at 4°C overnight. Subsequently, the membranes were incubated with the corresponding horseradish peroxidase-labeled secondary Abs at room temperature for 1 h. The chemiluminescence signal was obtained using a ChemiDox XRS system (Bio-Rad, USA). The densities of the bands were quantified with Quantity One Image software (Bio-Rad, USA).

### Vγ4^+^ γδ T-cell quantitative analysis

Vγ4^+^ γδ T cells were sorted from intraepithelial lymphocytes (IELs) as described previously [28]. Briefly, the ileal segment was collected and flushed out with PBS after mouse sacrifice, and then Peyer’s patches were removed. This segment was opened longitudinally and cut into 5 mm pieces. These pieces were incubated in a tube containing 2 mM EDTA and 4 mM 1,4-dithio-D-threitol in Ca- and Mg-free Hanks’ balanced salt solution for digestion. After that, the cell suspension was filtered through a nylon mesh filter and a column of glass wool. After Percoll gradient centrifugation, IELs were harvested in the cloudy interface area between 40 and 70% isotonic Percoll solution. The quantities of Vγ4^+^ γδ T cells were analyzed using an Attune acoustic focusing cytometer (Thermo Fisher, USA). Surface Abs were used as follows: anti-CD3 Ab, anti-TCR γδ Ab and anti-TCR Vγ4 Ab. For intracellular cytokine staining, cells were fixed, permeabilized and stained intracellularly for IL-17A. The relevant isotype control mAbs were also used.

### Cell culture

Intestinal epithelial cells (IECs, MODE-K) were purchased from ACTT and cultured according to the manufacturer’s instructions. Briefly, cells were plated and cultured in DMEM supplemented with 10% FBS, 100 U/ml penicillin and 100 μg/ml streptomycin at 37°C in a 5% CO_2_ incubator. We isolated and cultured Vγ4^+^ γδ T cells from the spleen because Vγ4^+^ γδ T cells from the intestine had poor survival and were easily contaminated. After removal from the organism, grinding, filtration, red blood cell lysis and centrifugation, multiple lymphocytes were placed together onto a 24-well plate, resulting in 1 × 10^6^ cells that were mixed in 500 μl of RPMI 1640 medium supplemented with 10% FBS, 2 ng/ml IL-2, 50 nm/ml mercaptoethanol, 1 μg/ml anti-CD28 Ab, 100 U/ml penicillin and 100 μg/ml streptomycin. Monoclonal Vγ4^+^ γδ T cells were generated with a plate coated with anti-Vγ4 Ab for 1 week. The number of Vγ4^+^ γδ T cells was confirmed by FACS analysis (purity requirement >80%). Finally, Vγ4^+^ γδ T cells were isolated by EasySep human γδ T-cell isolation.

### LCD treatment *in vitro*

Lipopolysaccharide (LPS) is widely used as an inflammatory agonist that can simulate postburn inflammation *in vivo* [29,30]. The concentration was determined in previous studies [25], and IECs and Vγ4^+^ γδ T cells underwent the same treatments: Control, LPS (50 μg/ml), and LPS (50 μg/mL) + LCD (10 μg/ml). After 12 h, Vγ4^+^ γδ T cells were isolated. The expression levels of Vγ4^+^ γδ T cells and IL-17A were detected by FACS analysis (Varioskan Flash, Thermo Fisher, USA).

### Patch–clamp experiment

The permeability of a single IEC membrane was measured by the patch–clamp technique in a conventional whole-cell configuration. All recordings were performed at 20–22°C using an Axopatch200B amplifier and Digidata1440A data acquisition system (Molecular Devices Inc., USA). Pipettes were fabricated from filament capillary glass using a P97 Micropipette Puller (Sutter Instrument Company, USA). Patch pipettes had typical resistances of 3–5 MΩ when fire-polished and filled with intracellular solution containing (in mM) 145 KCl, 1.2 CaCl_2_, 1.2 MgCl_2_ and 10 HEPES, pH 7.3, with KOH. The bathing solution contained serum-free DMEM. After the whole-cell configuration was established, the resting membrane potential was measured in “*I* = 0” mode. Data were saved for later analysis using clampfit 10.7 software.

### Paracellular permeability assay *in vitro*

The IEC monolayer was used to measure paracellular permeability. A FITC-labeled dextran (4 kDa) flux assay was conducted as previously described [31]. Briefly, the cells were seeded on PET polyester membrane transwell inserts (0.4 μm pore size) and allowed to cultivate for 2 days. The upper chamber was treated with FITC-labeled dextran (1 mg/ml) and then transwell inserts were incubated in the dark for 1 h. The amount of fluorescein permeation to the bottom of the chamber was measured with a fluorescence spectrophotometer (Varioskan Flash, Thermo Fisher, USA) at a wavelength of 480 nm/530 nm.

### ROS immunofluorescence detection

Immunofluorescence was performed as mentioned above. After being fixed and blocked, IECs were incubated with DCFH-DA (10 μmol/l) at 37°C in darkness for 30 min. Then, they were washed twice in PBS, and ROS were measured under a fluorescence microscope (BX53, Olympus, Japan) at excitation wavelengths of 488 nm and 525 nm.

### C‌CK-8 assay

IEC proliferation ability was evaluated using CCK-8. Cell suspensions were cultivated in a 96-well plate at 37°C in an incubator. A total of 10 μl of CCK-8 solution was added to the medium every 12 h until 72 h, and then the cell suspensions were incubated for an additional 1 h. The OD value (450 nm) was measured to assess cell proliferation.

### Scrape migration assay

IECs were seeded in a 6-well plate and grown to 90% confluence. Mitomycin C (10 μg/ml) was added to inhibit cell proliferation. Then, the cell monolayer was scraped with a 200-μl yellow pipette tip and washed in PBS. The scratch width was measured at 0 h. Subsequently, the cells were treated with LPS and/or LCD for 48 h. Images were obtained using a microscope (BX53, Olympus, Japan) at 0 and 48 h. The scratch width was quantified using ImageJ software.

### Statistical analysis

SPSS20.0 software (Chicago, USA) was used for data analysis. Continuous data are expressed as the mean ± SD and analyzed by independent-samples t tests and one-way ANVOA. Categorical data are presented as numbers and percentages, performed by chi-square test. *P* < 0.05 was considered to represent a statistically significant difference.

## Results

### Preparation of LCD nanoparticles

LCD nanoparticles were successfully synthesized by covalent conjugation between luminol and β-CD (Figure 1a). A circular granular shape of LCD nanoparticles was observed by transmission microscopy (Figure 1b). Moreover, LCD showed the physical characteristics of small size, with a diameter of 223.1 ± 1.4 nm, and zeta potential of −17.9 ± 1.3 mV (Figure 1c, d), which suggested these particles had the advantages of stable structure and the ability to be easily absorbed by the human body. All these data met the features and experimental requirements of a nanoparticle.

### LCD restores physical health and intestinal appearance

**Figure 2:**
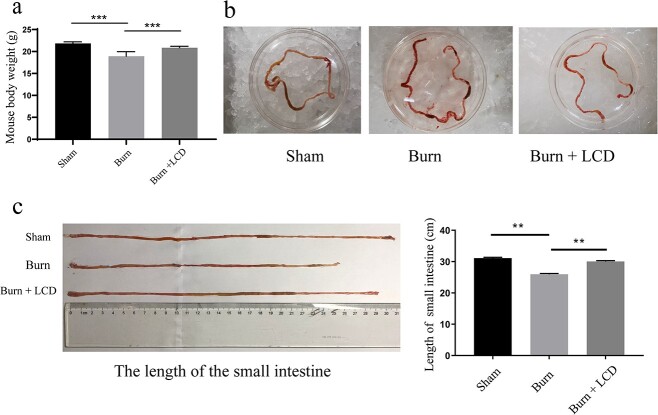
LCD nanoparticles restore physical health and intestinal appearance in a postburn mouse model. (**a**) Mouse body weight changes at seventh day. (**b**) Macroscopic views of the mouse small intestine. (**c**) Small intestine length changes. n = 5 in each group, ^**^p < 0.01 and ^***^p < 0.001. *LCD* luminol-conjugated cyclodextrin

At the seventh day after treatment, the body weight of mice decreased in the burn group, whereas the weight improved in the burn + LCD group, leading to a significant difference between these two groups (*p* < 0.001) (Figure 2a). Regarding intestinal appearance, the intestinal wall of the burn group appeared severely red and transparent, but this condition was alleviated in the burn + LCD group (Figure 2b). The length of the small intestine in the burn + LCD group was longer than that in the burn group (30.06 ± 0.24 *vs* 25.96 ± 0.24 cm, *p* < 0.01) (Figure 2c).

### LCD nanoparticles mitigate intestinal histopathological changes

**Figure 3:**
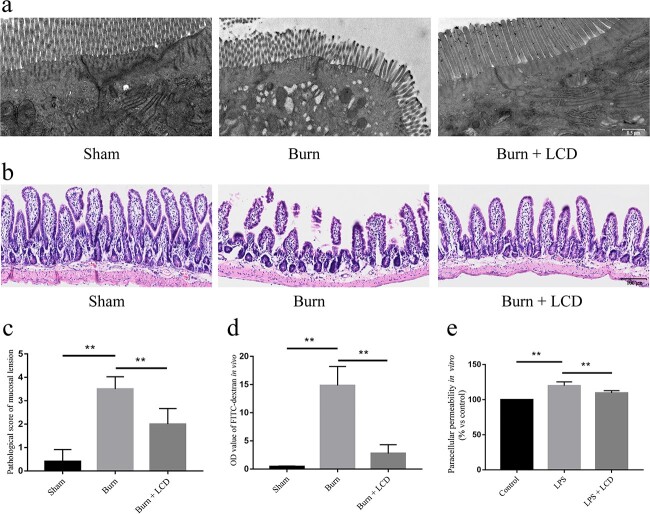
LCD nanoparticles repair intestinal mucosal damage and permeability. (**a**) TEM images of intestinal epithelial cells. Scale bar: 500 nm. (**b**) H&E-stain images and (**c**) mucosal lesion scores of the small intestine, scale bar: 100 μm. (**d**) Intestinal permeability and (**e**) paracellular permeability measurement by FITC-labeled dextran level. n = 5 in each group, ^**^p < 0.01. *LCD* luminol-conjugated cyclodextrin, *H&E* hematoxylin and eosin

Transmission electron microscopy photographs are shown in Figure 3a. The sham group exhibited clear and complete structures of TJs and organelles. However, the burn group exhibited a fuzzy TJ structure and profound organelle lesions with edema and vacuolar necrosis. In the burn + LCD group, the damage was mitigated compared to that in the burn group.

HE staining images are shown in Figure 3b. The sham group presented an approximately normal intestinal structure with few mucosal lesions and no inflammatory cell infiltration. However, the damage to the villi and submucosal tissue in the burn group was more serious than that in the sham group. The pathological scores of the two groups (burn group and burn + LCD group) were higher than those of the sham group, while the scores of the burn + LCD group were lower than those of the burn group (1.66 ± 0.68 *vs* 3.18 ± 0.72, *p* < 0.01) (Figure 3c).

### LCD nanoparticles recover intestinal permeability and increase TJ protein abundance

**Figure 4:**
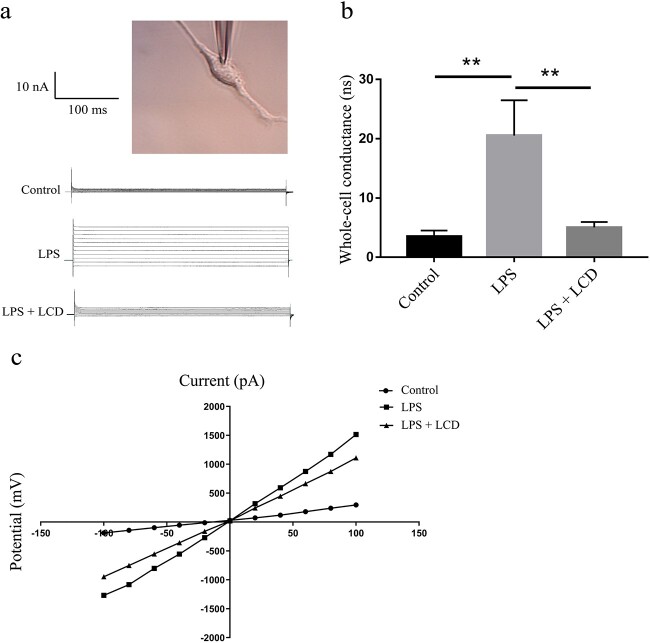
LCD nanoparticles alleviate membrane permeability by patch-clamp recording. (**a**) Representative experiment showing the whole cell currents in different groups. (**b**) Whole cell conductance changes. (**c**) Current (*I*)–voltage (*V*) relationship using data from (a). n = 5 in each group, and ^**^p < 0.01. LPS lipopolysaccharide, *LCD* luminol-conjugated cyclodextrin

The FITC-dextran assay is commonly used as an indirect measurement of intestinal permeability *in vivo* as well as paracellular permeability *in vitro*. Patch–clamp was used to measure the membrane permeability of a single cell. *In vivo*, FITC-dextran levels from plasma were increased in both the burn group and burn + LCD group compared to the sham group, while that parameter in the burn group was significantly higher than in the burn + LCD group (14.84 ± 3.39 *vs* 2.77 ± 1.55, *p* < 0.01) (Figure 3d). *In vitro*, LPS treatment significantly increased permeability, and LCD effectively reversed the outcomes caused by LPS stimulation. (100 *vs* 119.82 ± 5.64, *p* < 0.01; 109.57 ± 3.27 *vs* 119.82 ± 5.64, *p* < 0.01) (Figure 3e). The whole-cell-perforated basolateral membrane patches indicated that LPS stimulation increased whole-cell conductance from 3.51 ± 1.01 to 20.53 ± 5.95 nS, and the addition of LCD completely decreased whole-cell conductance from 20.53 ± 5.95 to 5.042 ± 0.93 nS (Figure 4a, b). The *I*–*V* curve suggested that LPS stimulation led to an increase in current, while LCD recovered the cell membrane permeability (Figure 4c). These findings suggested that LCD treatment can attenuate the increase in cell membrane penetrability induced by LPS.

**Figure 5:**
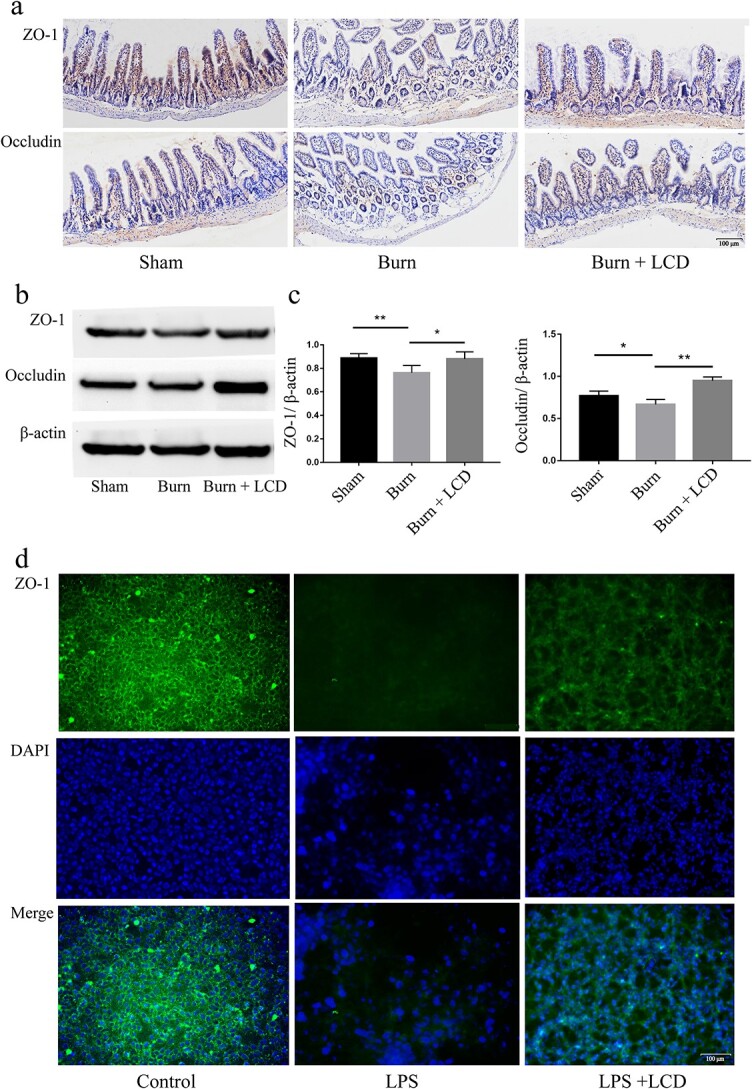
LCD nanoparticles elevate TJ protein abundance. (**a**) Immunohistochemistry assays indicating the expression of TJ proteins *in vivo*. (**b**) Western blotting and (**c**) quantitative analyses of ZO-1 and occludin. (**d**) Representative immunofluorescence images of ZO-1 alteration *in vitro*; green staining indicates ZO-1 and blue staining indicates nuclei. Scale bar: 100 μm, n = 5 in each group, ^*^p < 0.05 and ^**^p < 0.01. *ZO-1* zonula occludens-1, *LPS* lipopolysaccharide, *LCD* luminol-conjugated cyclodextrin, *DAPI* 4,6-diamino-2-phenyl indole, *TJ* tight junction

We next assessed the expression of ZO-1 and occludin, as altered TJ protein abundance is associated with changes in intestinal permeability. Immunohistochemistry, immunofluorescence and western blotting were performed. *In vivo*, ZO-1 in the burn group showed low intensity and discontinuous morphological distribution at the cell periphery, while the burn + LCD group exhibited a similar TJ protein expression pattern to the sham group, which showed abundant protein expression (Figure 5a). For immunoblotting, ZO-1 or occludin levels in the burn group were lower than those in the sham group but were partly reversed in the burn+LCD group (Figure 5b, c). *In vitro*, ZO-1 in the control group was distributed continuously at the periphery of cells, lying at the area of cell–cell junctions. The pattern of ZO-1 localization was disrupted in the LPS group, with loss of cell staining. However, ZO-1 staining in the burn + LCD group was similar to that in the control group, with a continuous straight line at the edge of the IECs (Figure 5d).

### LCD nanoparticles enhance IEC proliferation and migration and attenuate apoptosis

**Figure 6:**
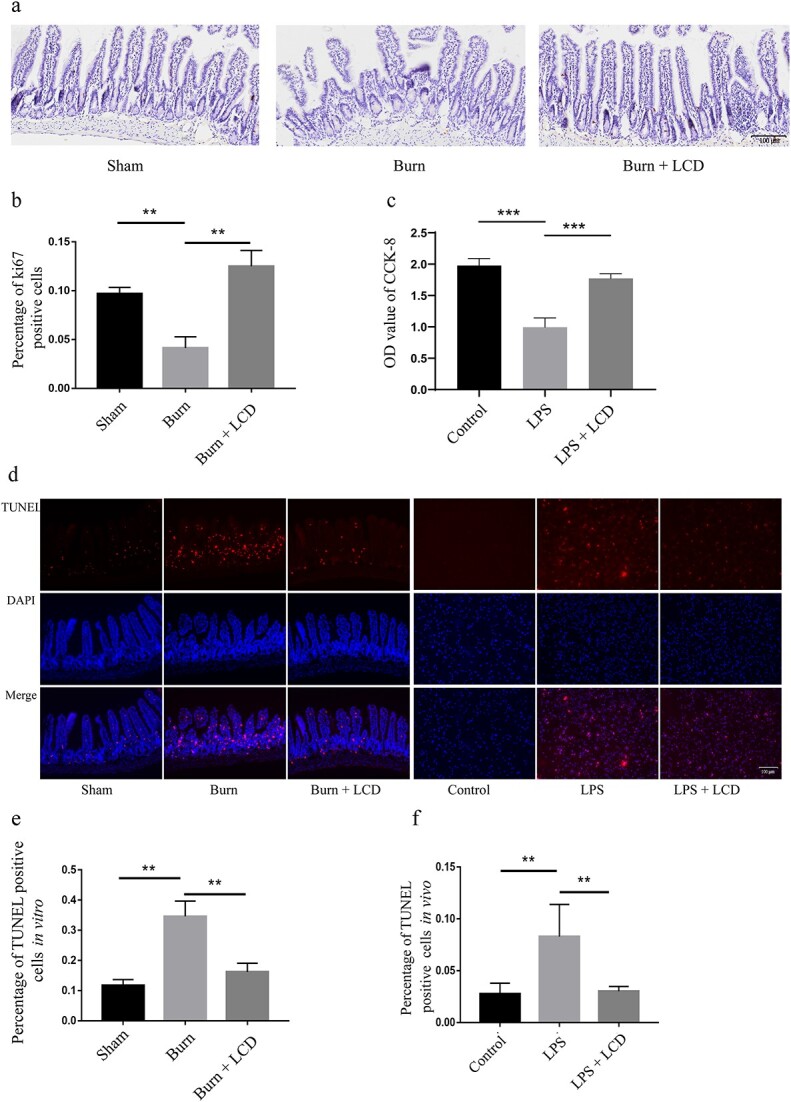
LCD nanoparticles enhance IEC proliferation and attenuate cell apoptosis. (**a**) Alteration of ki67-positive cells *in vitro* and (**b**) quantitative analyses. (**c**) CCK-8 assay showing IECs proliferation after 72 h *in vivo*. (**d**) Apoptotic activity by detecting the TUNEL-positive cells *in vitro* and *in vivo*; red staining indicates TUNEL and blue staining indicates nuclei. Quantitative analyses of TUNEL (**e**) *in vitro* and (**f**) *in vivo*. Scale bar: 100 μm. n = 5 in each group, ^**^p < 0.01 and ^***^p < 0.001. *LPS* lipopolysaccharide, *LCD* luminol-conjugated cyclodextrin, *DAPI *4,6-Diamino-2-phenyl indole, *TUNEL* terminal deoxynucleotidyl transferase dUTP nick-end labeling, *IEC*intestinal epithelial cells

IEC proliferation was estimated by ki67 staining and CCK-8 assays. *In vivo*, the rate of ki67-positive cells in the burn group (4.13 ± 1.15%) was markedly decreased compared with that in the sham group (9.69 ± 0.65%) (*p* < 0.01), while the percentage of proliferative cells in the burn + LCD group (12.51 ± 1.62%) was significantly higher than that in the Burn group (*p* < 0.01) (Figure 6a, b). The results of the CCK-8 assay *in vivo* were consistent with those *in vitro*, which indicated that LCD could enhance IEC proliferation when exposed to LPS (burn group 1.00 ± 0.15 *vs* burn + LCD group 1.77 ± 0.75 at 72 h, *p* < 0.01) (Figure 6c).

IEC apoptosis was estimated by TUNEL staining. *In vitro*, the rate of TUNEL-positive cells in the burn group (34.62 ± 5.06%) was markedly increased compared with that in the sham group (11.71 ± 1.93%) (*p* < 0.01), while the percentage of TUNEL-stained cells in the burn + LCD (16.18 ± 2.88) group was significantly decreased compared with that in the burn group (*p* < 0.01) (Figure 6d, e). The results of the *in vivo* experiments were consistent with those of the *in vitro* experiments, which suggested that LCD attenuated cell apoptosis after exposure to LPS (LPS group 8.29 ± 3.10% *vs* LPS + LCD group 3.02 ± 0.46%, *p* < 0.01 (Figure 6f).

### LCD nanoparticles inhibit the release of oxidative and inflammatory factors

**Figure 7:**
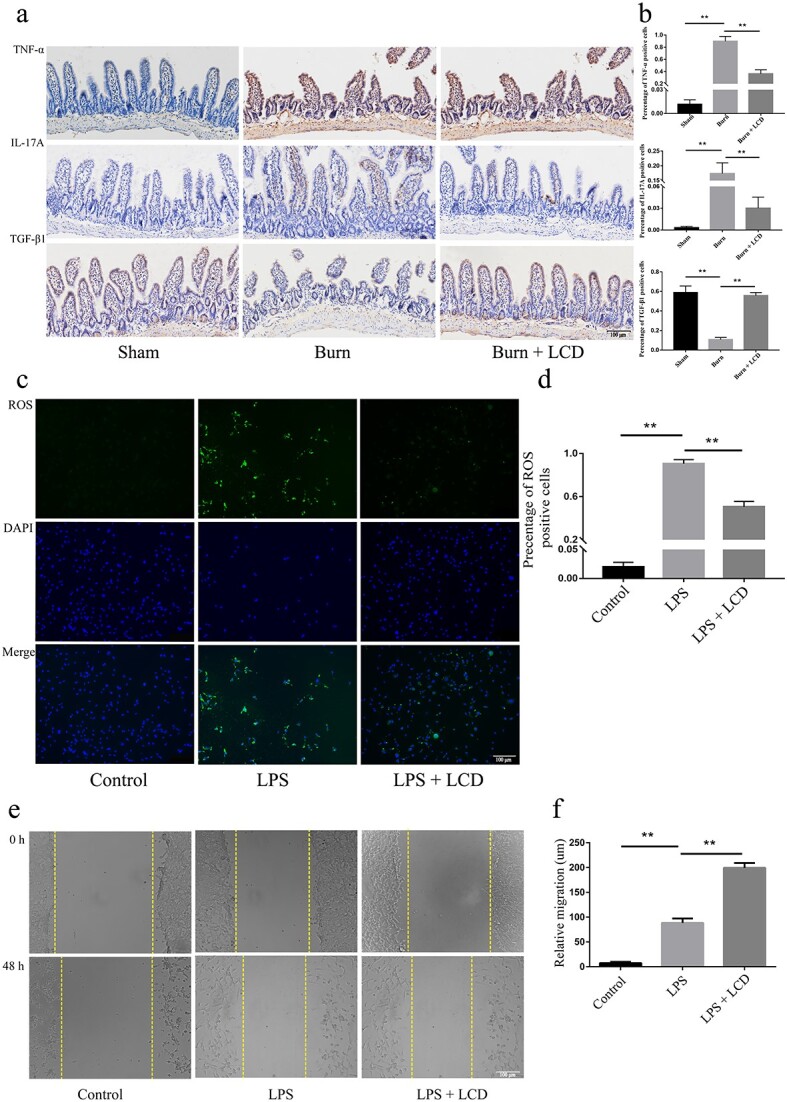
LCD nanoparticles inhibit the release of oxidative and inflammatory factors, boosting cell migration. (**a**) Immunohistochemistry assays and (**b**) quantitative analyses of cytokine proteins IL-17A, TNF-α and TGF-β, scale bar: 100 μm. (**c**) DCFH-DA images and (**d**) quantitative analyses of ROS; green staining indicates ROS and blue staining indicates nuclei, scale bar: 100 μm. (**e**) Scratch migration assay, and (**f**) quantitative analyses of migration. Scale bar: 200 μm. n = 5 in each group, ^**^p < 0.01. *LPS* lipopolysaccharide, *LCD* luminol-conjugated cyclodextrin, *DAPI* 4,6-diamino-2-phenyl indole, *ROS* reactive oxygen species

To explore the anti-inflammatory effect of LCD on burn conditions, the alterations in proinflammatory factors and anti-inflammatory factors (IL-17A, TNF-α and TGF-β) were analyzed by immunohistochemistry. The burn-treated intestine showed strong and diffuse immunoreactivities of IL-17A and TNF-α, but the TGF-β staining was weak. However, when mice were exposed to burns treated with LCD, IL-17A and TNF-α expression significantly decreased, and TGF-β increased (Figure 7a, b).

**Figure 8:**
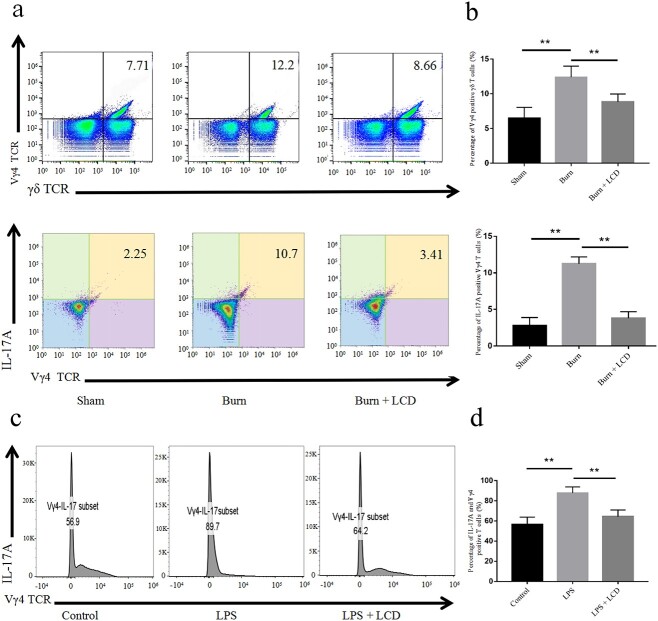
LCD nanoparticles reduce the level of IL-17A and Vγ4^+^ γδ T cells by cytometric analysis. (**a**) IL-17A and Vγ4 ^+^ γδ T cells isolated from IELs *in vivo* and (**b**) quantitative analyses. (**c**) IL-17A and Vγ4^+^ γδ T cells *in vitro* and (**d**) quantitative analyses. Stained with CD3, TCR γδ, Vγ4^+^ and IL-17A. n = 5 in each group, ^**^p < 0.01. *LPS* lipopolysaccharide, *LCD* luminol-conjugated cyclodextrin, *TCR* T cell receptor, *IL* interleukin, *IELs* intraepithelial lymphocytes

Intracellular and mitochondrial ROS in IECs were measured by a DCFH-DA assay. As shown in Figure 7c, d, there was increased ROS generation derived from cytoplasmic aggregates when IECs were exposed to LPS, while ROS levels decreased when they were supplemented with LCD, which suggested that LCD could inhibit ROS generation.

### LCD nanoparticles boosted IEC migration

A scratch migration assay was used to reflect cell migration ability and the balance of cell migration, proliferation and growth. LPS simulation shortened the width of the wound (88.53 ± 8.78 μm *vs* 7.50 ± 2.33 μm, *p* < 0.01). However, the wound width was reduced to a great degree with LCD treatment (199.5 ± 9.84 μm *vs* 7.50 ± 2.33 μm, *p* < 0.01). These data suggest that LCD increased IEC migration, which is important for intestinal barrier healing (Figure 7e, f).

### LCD nanoparticles inhibit the release of IL-17A from Vγ4^+^ γδ T cells

The results showed that the percentage of Vγ4^+^ γδ T cells was markedly increased in the burn group, but notably decreased in the burn + LCD group. Quantitative analysis of IL-17A production also showed the same trends (Figure 8a, b). When Vγ4^+^ γδ T cells were then isolated and treated with LCD, the level of IL-17A-positive cells decreased compared with that in the LPS group. These results indicate that LCD could inhibit the increase in Vγ4^+^ γδ T cells and IL-17A production (Figure 8c, d).

## Discussion

Severe burn injury directly leads to the disruption of intestinal barrier function, which contributes to the development of systemic inflammation and multiple organ failure. Traditional drugs, namely, steroidal anti-inflammatory drugs, are under no consideration for the multi-system damage they cause [32,33]. Due to the lack of a specific treatment, exploration of novel drugs for postburn intestinal damage is urgently needed. Nanoparticle treatment is a novel and powerful clinical strategy for treating intestinal diseases. Zeng reported that KPV (Lys-Pro-V al) peptide exerted anti-inflammatory effects by restoring inflamed epithelium functions [34]. Lee successfully synthetized hyaluronic acid–bilirubin nanomedicine to restore the epithelium barrier in acute colitis [35]. Despite the preliminary success of these anti-inflammatory nanotherapies, their complex structure, high cost and hydrophobicity of the nanoparticles render them inadequate for translational application. Herein, LCD nanoparticles have all the advantages of facile approach, hydrophilicity, biocompatibility and low cost. The β-CD part of LCD nanoparticles has been testified to inhibit the action of inflammatory mediators and T cells, thus mitigating inflammatory diseases [24,25]. In the present research, we explored the application of LCD nanoparticles for postburn intestinal therapy in a mouse model. Indeed, LCD nanoparticle treatment was shown to significantly decrease the symptoms of burn-induced intestinal injury, including body weight loss, mortality, shortening of the intestine, abnormalities of the intestinal appearance and histopathology. These data indicate that LCD nanoparticles have an efficient protective effect on burn-induced intestinal barrier disorder. Next, the potential mechanism of LCD nanoparticles for the treatment of burn-induced intestinal barrier injury was further studied, including their effect on the mechanical barrier, oxidative stress, inflammation and immunity.

The intestine barrier is pivotal for intestinal health, as its function is to resist the invasion of intraluminal bacteria, endotoxins and other pathogens [36]. The mechanical barrier is a crucial part of the intestinal barrier and is maintained through IECs and intercellular junctions [37,38]. TJs constitute the majority of intercellular junctions [39]. Severe burn injury leads to a mesenteric vasoconstriction and a lack of oxygen to the gut, resulting in mechanical intestinal barrier dysfunction, such as IEC apoptosis, loss of TJ structure and TJ proteins and increased intestinal permeability [40,41]. In this research, LCD nanoparticles demonstrated two strong capabilities in intestinal barrier recuperation. (1) IECs recovery: LCD nanoparticle treatment promoted IEC migration and proliferation, and inhibited IEC apoptosis. (2) TJ renewal: LCD nanoparticle treatment rehabilitated TJ structure and also increased the abundance of TJ proteins (ZO-1 and occludin). All components of mechanical barrier disruption could bring about increased intestinal and paracellular permeability, but LCD nanoparticle treatment restored it.

Oxidative stress is defined as the imbalance between the antioxidant and oxidative systems, bringing about ROS overdose [42–44]. Severe burns always initiate oxidative stress reactions and inflammation in the intestine [45,46], and an ROS overdose promotes the release of inflammatory cytokines, which can disturb intestinal barrier integrity [47]. Therefore, intervention aiming to inhibit ROS production is a critical step in reducing the progression of burn-mediated injury. In the present study, we cultured IECs with LCD nanoparticles *in vitro* and found lower ROS expression in the system compared to the control group. The results indicate that LCD nanoparticles possess anti-oxidative capacity in an inflammatory microenvironment, which indirectly explains their protective function as ROS suppressors in the maintenance of barrier integrity.

After severe burn, the resultant systemic inflammatory mediators interact to form a “cascade effect” network [48], causing further injures to the gut barrier and establishing a “vicious cycle” of inflammation [49]. Thus, inflammatory cytokine responses are an important pathophysiological factor that strongly reflect the development of burn-induced intestinal disorder. LCD nanoparticles’ anti-inflammatory effects have been verified in many inflammation-mediated organ models [25], and on these grounds, we have tried to apply this in a postburn gut model. In this study, our results confirmed that after LCD nanoparticle treatment, pro-inflammatory cytokine levels, like TNF-α and IL-17A, in the intestine were significantly suppressed, while levels of anti-inflammatory factors, like TGF-β, were increased, which was consistent with previously reported studies. The advantage of LCD nanoparticles for the inhibition of inflammation was again proved in the gut burn model.

γδ T cells, a major lymphocyte population with innate immune features, are located at epithelial surfaces of the intestine [50], where they serve as a key controller for the adaptive immune response. Significantly, γδ T cells act as an indispensable trigger in the early stage of inflammation. Recent studies have mentioned that γδ T cells can secrete IL-17A to amplify proinflammatory mediators in the early stages of inflammation [51–54]. To our knowledge, among all subtypes of γδ T cells, Vγ4^+^ is considered as the main IL-17A production candidate [55–57]. Li *et al*. reported that Vγ4^+^ γδ T cells were a major source of IL-17A in delayed wound repair [58]. Song *et al*. also detected that severe burns induced an increase in the proportion of Vγ4^+^ γδ T cells and IL-17A expression in the intestine of a mouse model [15]. In view of the above, we focused on the effect of LCD nanoparticles on Vγ4^+^ γδ T cells. We used flow cytometry and observed an increase in the percentage of Vγ4^+^ γδ T cells and IL-17A *in vivo* and *in vitro*, whereas LCD nanoparticles could significantly reverse this condition. The above data suggest that LCD nanoparticles could prevent Vγ4^+^ γδ T cells from activation and IL-17A secretion. Moreover, IL-17A secretion by Vγ4^+^ γδ T cells was regulated through nuclear factor-κB (NF-κB) or mammalian target of rapamycin (mTOR) signaling in most cases. O’Brien *et al*. reported that Vγ4^+^ γδ T cells did not make IL-17A in mice whose thymic epithelial cells lacked NF-κB-inducing kinase [59]. Cong *et al*. also indicated that Vγ4^+^ γδ T cells secreted IL-17A via regulating the PI3K/Akt/mTOR axis [60]. As was reported, LCD strongly modified NF-κB or the mTOR signaling pathway in different cell environments, including monocyte-endothelial adhesion [61], breast cancer cells’ resistance to TRAIL [62] and lipid raft-dependent activation in bronchial epithelial cells [63]. Hence, we inferred that LCD nanoparticles probably influence IL-17A secretion via signaling pathways mentioned above in Vγ4^+^ γδ T cells, which needs to be further investigated.

Mice were treated with LCD nanoparticles at a dose of 200 mg/kg via intravenous injection in *in vivo* experiments. However, in the *in vitro* experiments, the suitability of different doses of LCD nanoparticles was evaluated from 10 to 100 μg/ml. Our findings showed that LCD at both doses benefited intestinal barrier integrity and attenuated inflammation and oxidative stress, but the protective effect of LCD (10 μg/ml) was more obvious. We deduced that a low dose of LCD nanoparticles is more effective than a high dose. However, the optimal dose is still open to discussion.

One of this study’s limitations is that the various factors, such as LCD nanoparticle dosage, post-burn timepoint and burn degree, should be further considered and controlled. Moreover, the potential molecular mechanism of LCD nanoparticles’ function inside cells should be investigated in subsequent studies.

## Conclusions

To sum up, this study demonstrates the protective effect of LCD nanoparticles on severe burn-induced intestinal barrier breakdown. LCD nanoparticles promote intestinal mucous recovery by increasing cell proliferation, decreasing cell apoptosis and maintaining TJ proteins. These mechanisms are related to LCD’s abilities to scavenge ROS and suppress inflammation. Furthermore, we found that LCD nanoparticles effectively reduce the proportion of Vγ4^+^ γδ T cells and IL-17A, which may be one of the possible anti-inflammatory mechanisms. On the basis of this study, LCD nanoparticles facilitate a new type of nanotherapy for severe burn-induced intestinal barrier disruption.

## Abbreviations

CCK-8: Cell counting kit-8; β-CD: β-Cyclodextrin; CDI: Carbonyldiimidazole; DMEM: Dulbecco’s modified Eagle’s medium; DMF: Dimethylformamide; EDTA: Ethylenediaminetetraacetic acid; FBS: Fetal bovine serum; H&E: Hematoxylin and eosin; IECs: Intestinal epithelial cells; IELs: Intraepithelial lymphocytes; IL-17A: Interleukin-17A; LCD: Luminol-conjugated cyclodextrin; LPS: Lipopolysaccharide; mAb: Monoclonalantibody; mTOR: Mammalian target of rapamycin; NF-κB: Nuclear factor-κB; ROS: Reactive oxygen species; TEM: Transmission electron microscope; TJ: Tight junction; TNF-α: Tumor necrosis factor-α; TUNEL: Terminal deoxynucleotidyl transferase dUTP nick end labeling; ZO-1: Zonula occludens-1.

## Data Availability

Data will be made available on request.
