# Correction to: Luminol-conjugated cyclodextrin biological nanoparticles for the treatment of severe burn-induced intestinal barrier disruption

**DOI:** 10.1093/burnst/tkag014

**Published:** 2026-02-13

This is a correction to: Yajun Song, Yang Li, Wengang Hu, Feng Li, Hao Sheng, Chibing Huang, Xin Gou, Jingming Hou, Ji Zheng, Ya Xiao, Luminol-conjugated cyclodextrin biological nanoparticles for the treatment of severe burn-induced intestinal barrier disruption, *Burns & Trauma*, Volume 12, 2024, https://doi.org/10.1093/burnst/tkad054

The subfigure of the first row and third column in Figure Fig. 7a was incorrectly replaced with a duplicate of the same subfigure of the first row and second column during the formatting stage. This error was caused by improper file management during the collaborative process. This mistake does not have impact on the study's core findings, data validity, or conclusion.

There revised Figure 7 should read:


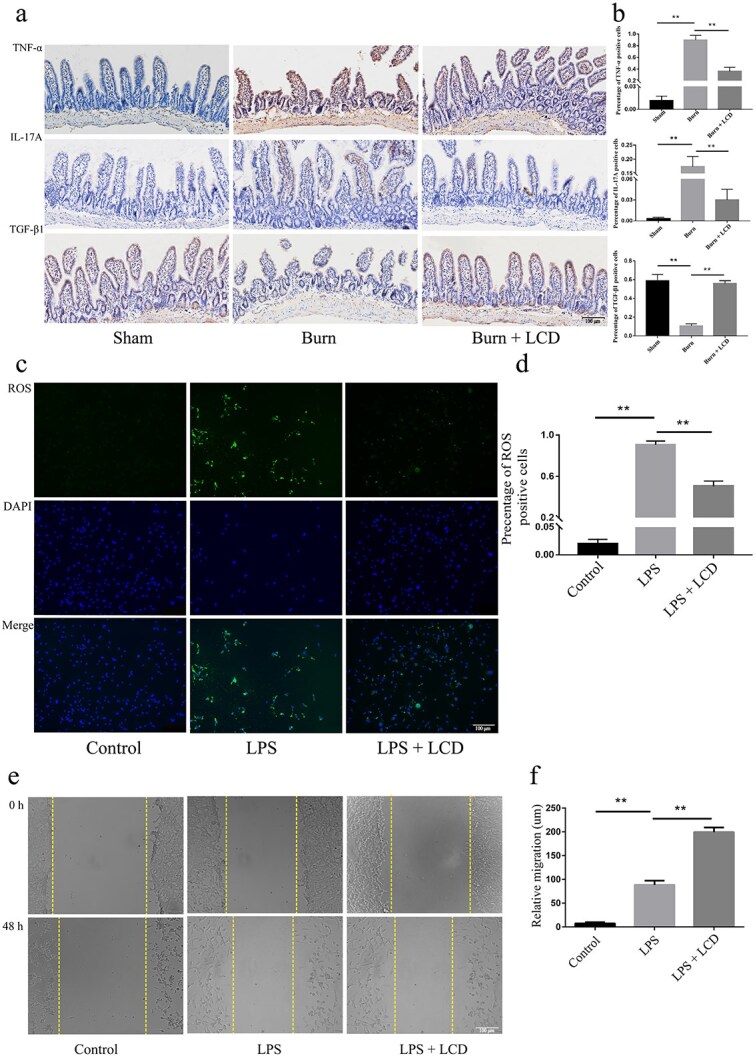


The errors have been outlined only in this correction notice to preserve the version of record.

